# Empirical Structure–Property Relationships of PLLA-*b*-PEG-*b*-PLLA Triblock Copolymers with Tunable Thermal, Tensile, and Swelling Behavior

**DOI:** 10.3390/polym18091127

**Published:** 2026-05-02

**Authors:** Yang Hu, Xiaoya Sun, Wei Wu, Adam K. Ekenseair

**Affiliations:** 1 Department of Chemical Engineering, College of Engineering, Northeastern University, Boston, MA 02115, USA; hu.yang@northeastern.edu (Y.H.); wu.wei@northeastern.edu (W.W.); 2 Department of Chemistry and Chemical Biology, College of Science, Northeastern University, Boston, MA 02115, USA; sun.xia@northeastern.edu

**Keywords:** polyethylene glycol, poly(L-lactic acid), copolymers

## Abstract

PLLA-*b*-PEG-*b*-PLLA triblock copolymers are promising materials because of their highly tunable properties. However, a systematic understanding of composition–property relationships remains limited. In this study, a series of A-B-A triblock copolymers was synthesized with polyethylene glycol (PEG) as soft center (B) domains and poly(L-lactic acid) (PLLA) as hard end (A) domains via ring-opening polymerization. Copolymer composition and molecular weights were characterized by proton nuclear magnetic resonance spectroscopy (^1^H NMR) and gel permeation chromatography (GPC). The thermal and mechanical properties of the copolymers were evaluated by differential scanning calorimetry (DSC) and tensile testing. We established quantitative structure–property relationships using empirical data, demonstrating that PLLA block length played a key role in modulating tensile properties, with a near-linear relationship, while PEG molecular weight critically influenced mechanical stability. An approximate minimum PLLA block length of 20 repeat units was found as a threshold required to maintain structural integrity during in vitro 24 h swelling. These findings provide insights and practical guidance for the design of triblock copolymers with tunable thermal, mechanical, and swelling properties of PLLA-*b*-PEG-*b*-PLLA triblock copolymers.

## 1. Introduction

Amphiphilic polymers have been investigated for the past few decades because of their versatile potential in various applications [1,2,3,4]. Among multiple types of amphiphilic polymers, ABA-type triblock copolymers play an active role. Copolymerizing both hydrophobic and hydrophilic materials into a copolymer material constitutes a promising method for modulating the basic properties of each homopolymer. Through variation in the ratio of PEG and PLLA, the limitations of purely hydrophilic or hydrophobic materials can be overcome, allowing precise control over copolymer properties for specific uses [5,6].

Poly(L-lactic acid) (PLLA) is a biodegradable polymer [7,8] that naturally degrades to lactic acid, which is then metabolized into water and carbon dioxide in humans [9]. In 2004, the Food and Drug Administration (FDA) approved an injectable implant (Sculptra^®^, Zahlerweg 10, Zug, Switzerland) containing PLLA for the treatment of lipoatrophy in patients with human immunodeficiency virus to restore the loss of facial volume [10]. Based on this approval, many PLLA-based medical devices have been developed for various clinical uses [11,12]. Poly(ethylene glycol) (PEG) is a class of hydrophilic polymers that are biocompatible [13] and were approved by the FDA in 1999 [14]. To date, PEG itself and its modification, PEGylation, have been widely used in the pharmaceutical industry due to their well-established safety [15,16,17]. For example, PEG 400 is commonly used as a pharmaceutical solvent in eye drops and as a humectant [18]. PEG 3350 is used as an osmotic laxative to treat occasional constipation by drawing water into the bowel [19].

Various studies have shown that PLLA-PEG-PLLA triblock copolymers comprising hydrophilic PEG and hydrophobic PLLA have highly tunable properties [20,21]. By individually varying the wt% of PEG or PLLA, the crystallization and melting behavior of the copolymer are controlled by both crystalline PLLA and PEG domains in the triblock structures [22]. In both amorphous and crystallized copolymers, when the molecular weight of the PLLA domain is higher than that of the PEG domain, PLLA-PEG-PLLA copolymers showed thermally phase-separated behaviors [23]. The Young’s modulus, yield strength, and elongation at break were enhanced and directly correlated with the increasing amount of PLLA [23]. With an increasing amount of L-lactide in the reaction system, the molecular weight of PLLA-PEG-PLLA copolymers increased while the hydrophilic weight fraction decreased [24]. Polymerizing PEG with PLLA overcomes the drawbacks of the hydrophilic nature of PEG, meanwhile utilizing the hydrophobic property of PLLA with non-toxic degradation products. Hydrogels made of PLLA-PEG-PLLA with comparable compositions exhibited similar swelling ratios of approximately 4 [25], as well as those made of multi-arm PEG [26]. Despite extensive studies on PLLA-PEG-based copolymers, a systematic understanding of how block composition simultaneously governs thermal, mechanical, and swelling behaviors remains limited.

In this study, a range of molecular weights of PEG (1450, 3000, 8000, and 20,000 g/mol) was used to study its impact on the thermal, physical, and mechanical properties of the copolymers. L-lactide and PEG were used as monomers, and PEG was also used as a macroinitiator. By varying both PEG and PLLA block lengths, a series of PLLA-*b*-PEG-*b*-PLLA copolymers was synthesized with varied A (PLLA) and B (PEG) block lengths via ring-opening polymerization. Chemical compositions were characterized. The thermal, mechanical, and swelling behaviors of the copolymers were evaluated. We demonstrated that there were empirical linear correlations of copolymer composition to its material properties. Key thresholds in block length that govern structural integrity and performance were identified, providing a rational design framework for triblock copolymers.

## 2. Materials and Methods

### 2.1. Materials

Pharmaceutical-grade poly (ethylene glycol) (PEG; M_n_ = 1450, 3400, 8000, or 20,000 kDa) was purchased from Polysciences, Inc. (Warrington, PA, USA) and used as received. 3,6-Dimethyl-1,4-dioxane-2,5-dione (L-lactide), Tin (II) 2-ethylhexanoate (stannous octoate), HPLC-grade methanol and diethyl ether were purchased from Sigma-Aldrich (Sigma, St. Louis, MO, USA) and used as received. HPLC-grade toluene was purchased from Sigma-Aldrich (Sigma, St. Louis, MO, USA) and dried for 3–4 days prior to use with molecular sieves (4 Å, 4–8 mesh, Sigma, St. Louis, MO, USA). HPLC-grade dichloromethane was purchased from EMD Millipore Corporation (EMD Millipore Corp, Darmstadt, Germany). Biopsy punches were purchased from Integra LifeSciences (Princeton, NJ, USA).

#### Synthesis of Poly(L-lactic acid)-*b*-(polyethylene glycol)-*b*-poly(L-lactic acid) (PLLA-*b*-PEG-*b*-PLLA) Triblock Copolymers

PLLA-*b*-PEG-*b*-PLLA copolymers with varied block lengths were synthesized through ring-opening polymerization. In a typical reaction, 1.25 g of PEG was first dissolved at 10% *wt*/*v* in dried toluene at 60 °C under a nitrogen atmosphere overnight. Then, 0.45, 0.89, 1.78, or 2.56 g of L-lactide, depending on the desired PLLA block length (A-D, respectively), was dissolved at 10% *wt*/*v* in dried toluene and combined with the PEG solution. Tin (II) 2-ethylhexanoate was added as a catalyst at 5 wt% of the total monomer content, and the reaction mixture was continuously stirred at 140 °C under a nitrogen environment for 14 h. The product was then cooled down to room temperature, concentrated through rotary evaporation (BUCHI, Newcastle, DE, USA), re-dissolved in 10% *w*/*v* dichloromethane, and precipitated into at least 10-times excess of cold diethyl ether to remove unreacted monomer and low molecular-weight oligomers. After filtration, the precipitate was stored *in vacuo* at room temperature overnight to yield fine white powders.

### 2.2. Methods

#### 2.2.1. Proton Nuclear Magnetic Resonance (^1^H NMR) Spectroscopy

^1^H NMR spectra were obtained using a 500 MHz spectrometer (Varian Inova, CA, USA). Sample materials were dissolved in deuterated chloroform (CDCl_3_) (typical concentration: 20 mg/mL) containing 0.05 wt% 3-(trimethylsilyl) propionic-2,2,3,3-d_4_ acid sodium salt (TSP) as an internal shift reference (Sigma-Aldrich, St, Louis, MO, USA). The characteristic methylene protons of PEG were observed at *δ* ≈ 3.64 ppm as a reference peak; methine and methyl protons of PLLA were detected at *δ* ≈ 5.15 ppm and *δ* ≈ 1.58 ppm, respectively. The degree of polymerization of PLLA blocks (DP_PLLA_) was determined using the integration ratio of the PLLA methine-to-PEG methylene peak. The number-average molecular weight (M_n_) of the copolymers was calculated based on the obtained PLLA block length and the known molecular weight of PEG. MestReNova (version 16.0) was used to analyze and integrate the NMR spectra for peak assignment and the quantitative determination of polymer composition.

#### 2.2.2. Gel Permeation Chromatography (GPC)

Molecular weight distributions of PLLA-*b*-PEG-*b*-PLLA copolymers were determined by GPC (TOSOH EcoSEC HLC-8320, King of Prussia, PA, USA). Samples were prepared in the mobile phase (80% methanol/20% water, *v*/*v*) at a concentration of 1 mg/mL and sterile-filtered prior to analysis. The number-average molecular weight (M_n_), weight-average molecular weight (M_w_) and poly dispersity index (PDI = M_w_/M_n_) were determined in triplicate relative to narrowly dispersed PEG standards (Polymer Standards Service-USA, Inc, Amherst, MA, USA). Super AWM-H and Super AW 3000 columns (Tosoh Bioscience LLC, King of Prussia, PA, USA) were used in series for separation. A series of PEG standards (982, 3020, 6690, 9890, 18,600, 44,000, and 69,100 g/mol) was used for calibration, with corresponding approximated results of 982, 3040, 6543, 10,093, 18,583, 43,711, and 69,341 g/mol. The mass percent of PEG (PEG wt%) in each sample was calculated using the ratio of PEG M_n_ to the total M_n_ of each sample.

#### 2.2.3. Differential Scanning Calorimetry (DSC)

The thermal properties—onset (T_m,onset_), peak melting temperatures (T_m,peak_), and melting enthalpy of the copolymers—were determined by DSC. Samples were manually ground into a fine white powder; approximately 30 mg of the sample was placed into a Tzero hermetic aluminum DSC pan (TA Instruments, Newcastle, DE, USA) and capped using TA Instruments Tzero Press (TA Instruments, Newcastle, DE, USA). Thermograms were recorded in triplicate for each sample using a TA Instruments DSC Q2000 (TA Instruments, Newcastle, DE, USA) with a refrigerated cooling system. A thermogram of the same empty pan was used as a reference. Heat–cool–heat procedures were utilized to eliminate the thermal history of individual samples. In a typical run, the oven was equilibrated at 40 °C for 1 min, heated to 100 °C at a rate of 5 °C/min, held for 1 min, cooled down to 0 °C at a rate of 5 °C/min, held for 1 min, and finally heated to 100 °C at a rate of 5 °C/min. Universal Analysis 2000 software (version 24.11, TA Instruments, Newcastle, DE, USA), provided by the DSC equipment, was used to quantify the thermal properties of each replicate. The melting enthalpy of the sample (∆H) was measured by integrating the endothermic peak in the second heating cycle based on the mass of the individual sample. The melting enthalpy of pure PEG in different M_n_ was measured by integrating the second endothermic peak. The relative melting enthalpy of PEG in each copolymer (∆H_PEG_) was calculated using Equation (1):

(1) $${∆H}_{PEG}=\;\frac{∆H}{PEG\;wt\%}$$

The crystallinity of each pure PEG was assumed as 100%, and the relative crystallinity to PEG (X_PEG_) was calculated using Equation (2):

(2) $$X_{PEG}=\;\frac{∆H}{∆H_{PEG}}\;\times \;100\%$$

#### 2.2.4. Tensile Test

The mechanical properties of copolymers were evaluated by stress, strain, and Young’s modulus. Samples were molded in triplicate by extruding melted copolymers (5~10 min at 80 °C) using glass syringes into a Teflon mold (20 mm in length, 5 mm in width, and 2 mm in height). Such samples were cooled down to room temperature (approximately 20 °C) for at least 30 min. Extra outlined parts were trimmed off using steel blades (Thermo Fisher Scientific, Waltham, MA, USA) to obtain a cuboid shape. Cuboid samples were placed under vacuum conditions at room temperature for at least 24 h prior to tensile testing. Prior to tensile testing, all samples were pre-tested using the manual bend test to exclude easily broken samples. Tensile testing was performed using ElectroForce 3200 (TA Instruments, Newcastle, DE, USA). After calibration, samples were placed between two aligned axial clamps with a gauge dimension of 10 mm in length and 5 mm in width. Samples were continuously stretched to failure at a tensile speed of 5 mm/min. The initial slope (0.2%) of the engineering stress versus the engineering strain curve was used to determine Young’s modulus.

#### 2.2.5. 24 h In Vitro Swelling

Copolymer powders were loaded into glass syringes and heated at 80 °C for 5 min in an oil bath. The melted copolymer solution was manually extruded between two flat Teflon molds with a 1 mm gap. Samples were cooled down *in situ* at room temperature overnight. Disk-shaped samples (diameter: 6 mm; thickness:1 mm) were made using a biopsy punch. The initial dry mass of each individual disk was recorded as m_dry_. Each disk was placed in an empty scintillation vial and immersed in 5 mL of PBS solution for 24 h. The PBS solution was removed; the mass of swollen samples was recorded as m_sw_. The swelling ratio, q, was calculated as q = m_sw_/m_dry_. Three individual samples were used for each experimental condition.

### 2.3. Statistics

All experiments were performed in triplicate, and results were expressed as means ± standard deviation. Data analysis was performed using GraphPad Prism software (version 10.6.1). To compare T_m,onset_, T_m,peak_, and melting enthalpy, a one-way analysis of variance (ANOVA) test with Tukey’s post hoc test was used. To compare ultimate stress, ultimate strain, and Young’s modulus, a two-way analysis of variance (ANOVA) test with Tukey’s post hoc test was used. To study the linearity of PLLA mol % to T_m,onset_ and T_m,peak_, and PLLA block length to Young’s modulus, a simple linear regression was performed using GraphPad Prism (version 10.6.1). Best-fit slopes and intercepts were used to plot a linear regression curve. Goodness of fits (r^2^) were reported in each curve. Statistical significance is 95%.

## 3. Results

### 3.1. Triblock Copolymer Synthesis and Characterization

**Figure 1:**
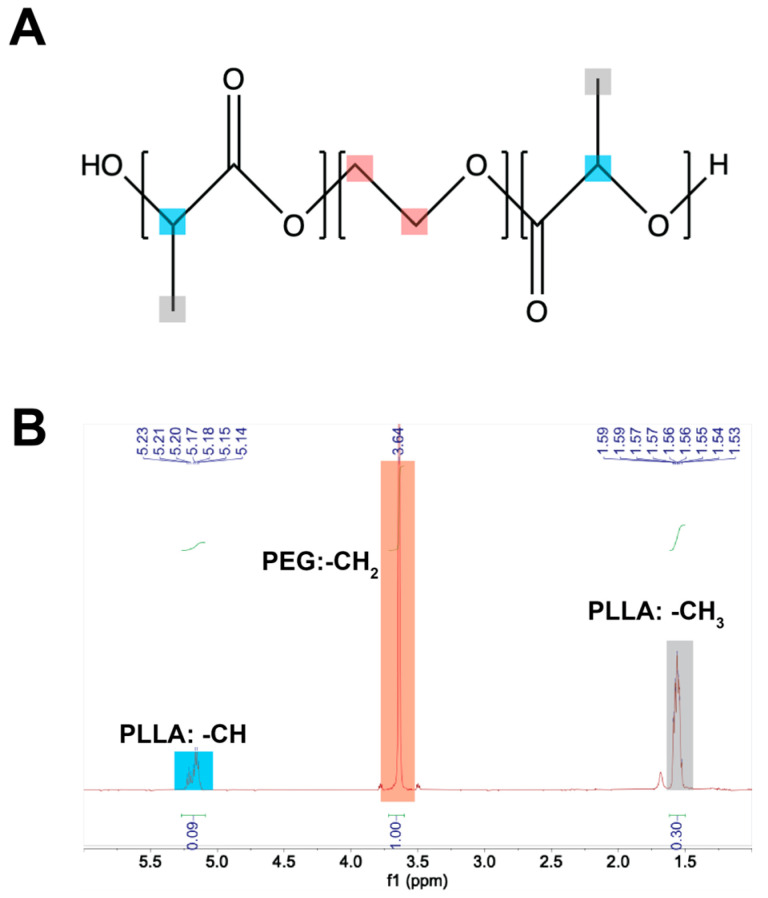
Representative ^1^H NMR spectrum of PLLA-*b*-PEG-*b*-PLLA triblock copolymers (PEG Mn ≈ 20 kDa) recorded using CDCl_3_. (**A**) Chemical structure of PLLA-*b*-PEG-*b*-PLLA triblock copolymers. (**B**) Example ^1^H NMR spectrum of PEG 20k-C.

PLLA-*b*-PEG-*b*-PLLA triblock copolymers were synthesized by ring-opening polymerization in the presence of PEG as an initiator. Increasing lengths of PLLA side blocks were successfully polymerized onto PEG center blocks, which was confirmed by ^1^H NMR (Figure 1). The example spectra of the PEG 20k-C sample showed three key resonances (PLLA-CH_3_: 1.8 ppm, PEG-CH_2_: 3.6 ppm; PLLA-CH: 5.3 ppm). These three resonances were unique, confirming that copolymer composition can be directly determined by comparing the integral areas of the methine group on PLLA and the methylene group on PEG. The number average molecular weight (M_n_), mol % PLLA, M_n_ ratio of PEG to PLLA, and the average PLLA block length on each side were summarized in Table 1 for each copolymer composition.

**Figure 2:**
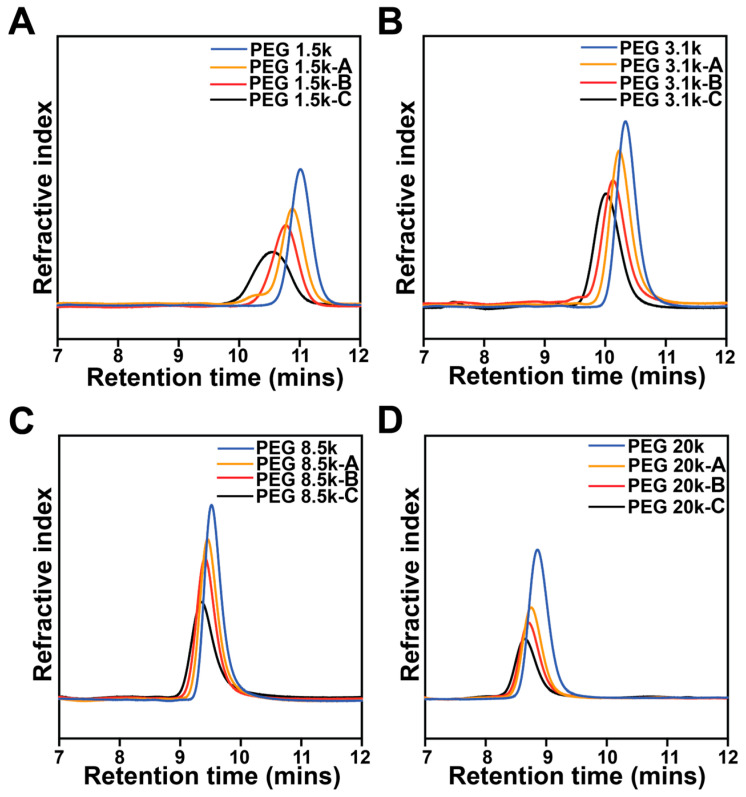
GPC spectra of triblock PLLA-*b*-PEG-*b*-PLLA copolymers with varied PEG molecular weights and PLLA side blocks. Refractive index was plotted as a function of retention times. (**A**) PEG 1.5k, (**B**) PEG 3.1K, (**C**) PEG 8.5k, and (**D**) PEG 20k with their corresponding modifications of varied PLLA contents.

The molecular weights of all copolymers were further evaluated by GPC (Figure 2, Table 1). For each set of copolymers with the same PEG, the molecular weight of each composition increased with increasing PLLA block length. The spectra shifted toward the direction of lower retention times, indicating that larger copolymers were eluted at earlier time points. GPC results generally agreed with ^1^H NMR results, while the M_n_ of PEG 20k-B and 20k-C were comparable. Most samples showed successful and controlled ring-opening polymerization in GPC analysis, with narrow PDI. PEG 20k-D (PLLA_220.0_-PEG_452_-PLLA_220.0_) was completely insoluble in the GPC mobile phase (methanol/water 80/20 *v*/*v*) after vigorous mixing using a vortex for an hour and on a shaker overnight, showing the hydrophobicity of PLLA domains. For the group of PEG 20k, we observed a growing mismatch of the M_n_ determined by ^1^HNMR and GPC with increasing PLLA block length. Similar discrepancies between GPC and ^1^HNMR results in increasing molecular weights were similarly observed by Cui et al. [27] and Mao et al. [28]. In the scope of this study, it may be attributed to the lack of PEG standards with a M_n_ of 20,000 g/mol. To be consistent in representing repeat units of PEG and PLLA, the results from the ^1^H NMR characterization were reported as in previous studies [29,30].

### 3.2. Thermal Properties

**Figure 3:**
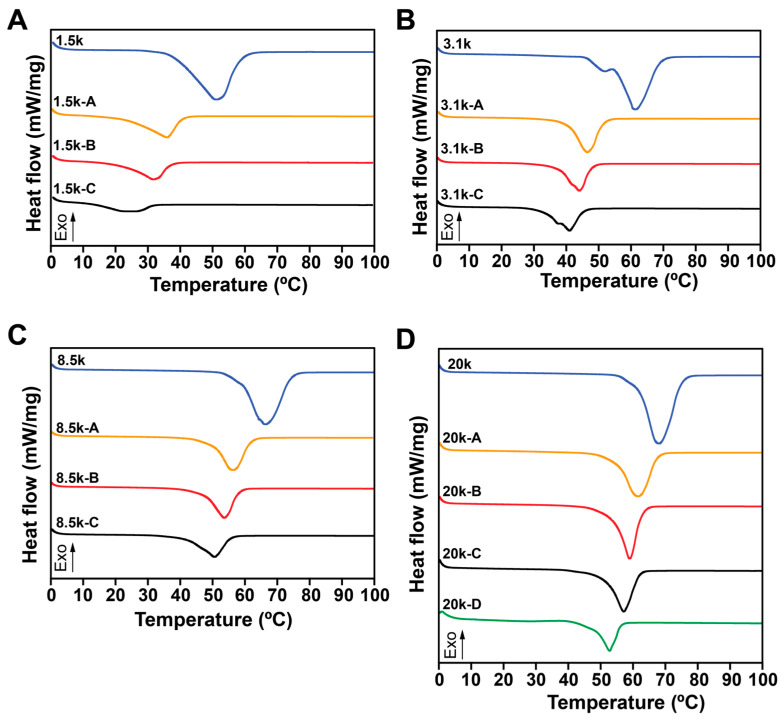
DSC heating curves of triblock PLLA-*b*-PEG-*b*-PLLA copolymers with PEG center blocks of (**A**) 1.5 kDa, (**B**) 3.1 kDa, (**C**) 8.5 kDa, and (**D**) 20 kDa.

Thermal properties of all copolymers were determined by DSC. The full thermal spectra of all samples are shown in Figure S1A–D. To inspect whether L-lactide was homopolymerized to PLLA during the ring-opening polymerization and the aggregation of PLLA domains, we purposely processed PEG 20k-D at a heating and cooling rate of 10 °C/min from 0 °C to 200 °C [31] (Figure S1E). This sample had the highest amount of L-lactide added to the reaction system and the highest number of PLLA repeat units. The typical endothermic peak of PLLA was not observed at the melting point of PLLA (175–180 °C). The exothermic peak was likely due to the specific test rate, which meant that the crystallization was incomplete during the cooling cycle. Thus, 100 °C was determined as the endpoint during the heating cycle for all samples. To be consistent, a heating and cooling rate of 5 °C/min was used for all samples, and the spectra are shown in Figure 3. Notably, the endothermic peak of the PEG 1.5k-C sample was almost flat, indicating that the addition of PLLA significantly interrupted the crystallinity of the PEG domain (Figure 3A). With an increasing molecular weight of PEG, endothermic peaks were noticeable (Figure 3B–D).

**Figure 4:**
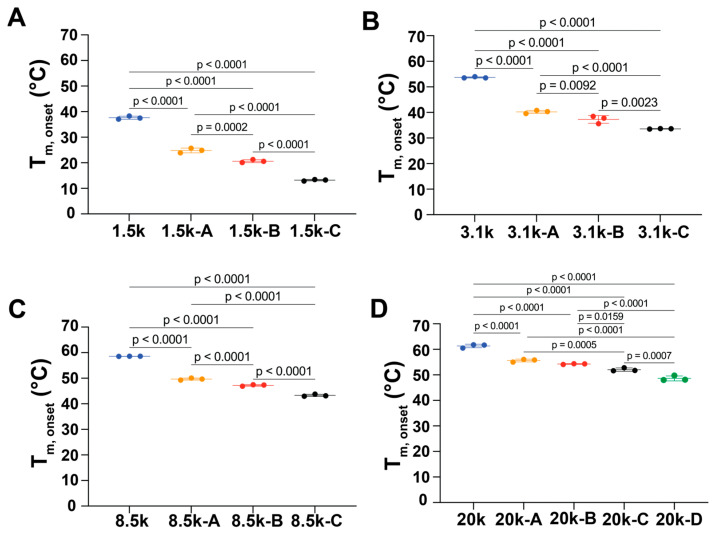
Onset melting temperatures (T_m,onset_) of triblock PLLA-*b*-PEG-*b*-PLLA copolymers with PEG center blocks of (**A**) 1.5 kDa, (**B**) 3.1 kDa, (**C**) 8 kDa, and (**D**) 20 kDa. One-way ANOVA tests with Tukey’s test as a post hoc test were used. Some error bars are small on the plot and can not be distinguished. Error bar: standard deviation.

Onset melting temperature (T_m,onset_) is the temperature at which the crystalline domains of the copolymer start to melt. Pure PEG displayed the highest T_m,onset_ across all corresponding samples that were polymerized with PLLA (Figure 4). During the heating and cooling cycle, these PEG domains were fully self-crystallized to form lamellar structures. For pure PEG samples, the T_m,onset_ increased from 37.6 ± 0.6 °C of PEG 1.5k to 61.3 ± 0.7 °C of PEG 20k (Figure 4, Table 2), indicating that an increase in the molecular weight of PEG monomers increased the crystallinity of PEG domains.

By adding an increased mass of L-lactide to the reaction system, the T_m,onset_ of samples at each experimental condition significantly dropped (Figure 4). In each group of copolymers with the same molecular weight of PEG, samples with the longest PLLA block lengths displayed the lowest T_m,onset_. This observation indicated that the addition of PLLA domains interrupted the formation of crystalline PEG, thus decreasing the T_m,onset_. This finding was consistent with our observation when preparing DSC samples, where we found that in the group of copolymers with PEG 1.5k, all copolymer samples (PEG 1.5k A–C) were in a liquid state at room temperature (~25 °C). All other samples were in solid states when preparing samples for DSC tests.

**Figure 5:**
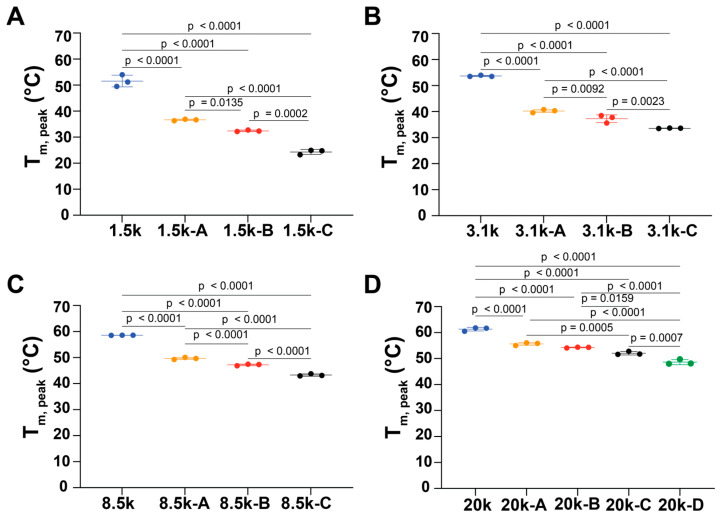
Peak melting temperatures (T_m,peak_) of triblock PLLA-*b*-PEG-*b*-PLLA copolymers with PEG center blocks of (**A**) 1.5 kDa, (**B**) 3.1 kDa, (**C**) 8.5 kDa, and (**D**) 20 kDa. One-way ANOVA tests with Tukey’s post hoc test were used. *p*-values for each comparison are shown above the line. Some error bars are small on the plot and cannot be distinguished. Error bar: standard deviation.

Peak melting temperature (T_m,peak_) is the temperature at the maximum of the endothermic peak in DSC. Similarly to T_m,onset_, T_m,peak_ significantly decreased with the addition of PLLA domains (Figure 5). For the initial addition of PLLA to the PEG block of each condition, T_m,peak_ decreased by ~15 °C for samples of PEG 1.5k and 3.1k, ~10 °C for samples of PEG 8.5k, and ~8 °C for samples of PEG 20k (Table 2). This indicates that the thermal stability of the main crystals in each type of copolymer progressively decreased.

**Figure 6:**
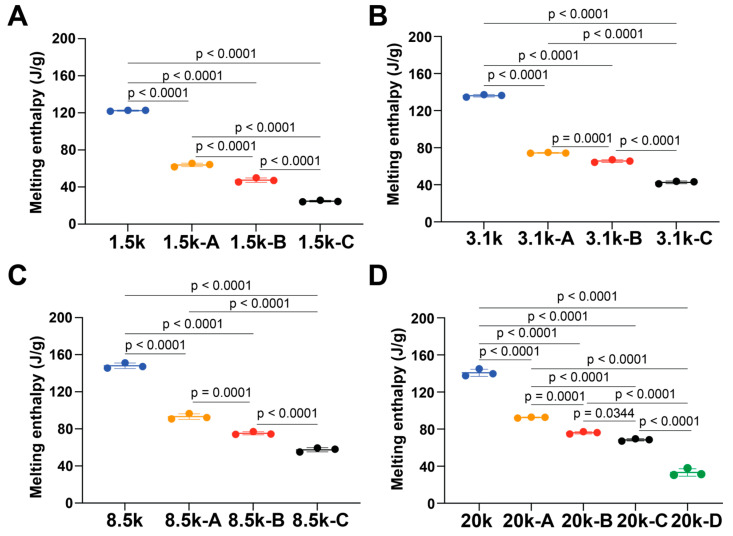
Melting enthalpy (∆H) triblock PLLA-*b*-PEG-*b*-PLLA copolymers with PEG center blocks of (**A**) 1.5 kDa, (**B**) 3.1 kDa, (**C**) 8 kDa, and (**D**) 20 kDa. One-way ANOVA tests with Tukey’s post hoc test were used. *p*-values for each comparison are shown above the line. Some error bars are small on the plot and cannot be distinguished. Error bar: standard deviation.

Melting enthalpy (∆H, J/g) is the amount of heat absorbed by a material during the melting process; it was measured by integrating the area of the endothermic peak in the heating cycle. Compared to each pure PEG, the energy required to melt crystalline domains of copolymers to convert them to an amorphous state was significantly decreased (Figure 6). Two samples with significantly different molecular weights, PEG 1.5k-C and PEG 20k-D, had comparable ∆H (Table 2, PEG 1.5k-C: 24.8 ± 0.9 and PEG 20k-D: 33.3 ± 4.0 J/g). Although the PLLA block lengths are very different, this similarity may be due to the M_n_ ratio of PEG to PLLA.

### 3.3. 24 h In Vitro Swelling

**Figure 7:**
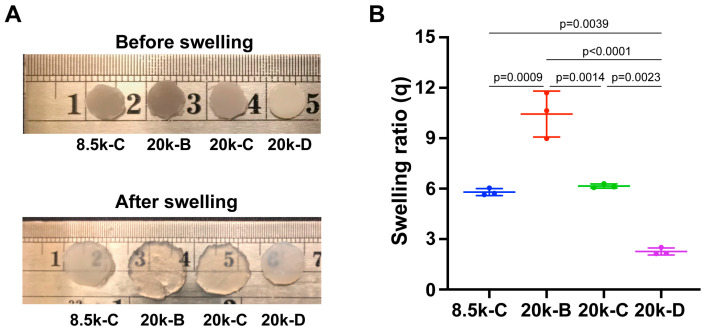
Weight swelling ratio (q) of copolymer samples with varied PEG molecular weight (8.5 kDa and 20 kDa) and increasing PLLA block length after 24 h. (**A**) Samples before and after swelling. (**B**) Swelling ratio. Two-way ANOVA test with Tukey’s post hoc test was used. Error bar: standard deviation.

We investigated the degree of swelling of triblock copolymers. Disk-shaped samples of all copolymers were individually incubated in excess PBS solution for 24 h at 37 °C. After 24 h, the structural integrity of all samples was evaluated visually. Samples with PEG molecular weights of 1.5 kDa and 3.4 kDa were completely dissolved into clear solutions. PEG 8.5k-A, 8.5k-B, and PEG 20k-A did not fully dissolve but instead disintegrated into small fragments, producing cloudy solutions or visible particulates. The aforementioned samples were excluded from the in vitro swelling test. For the remaining samples, disk samples before and after swelling are shown in Figure 7A. PEG 20k-B had the highest swelling ratio (q_20k-B_ = 10.4 ± 1.4), PEG 8.5k-C and PEG 20k-C had intermediate swelling ratios (q_8.5k-C_ = 5.8 ± 0.2, q_20k-C_ = 6.2 ± 0.1; *p* = 0.9269), and PEG 20k-D had the lowest swelling ratio (q_20k-D_ = 2.3 ± 0.2) (Figure 7).

### 3.4. Mechanical Properties

Tensile failure tests were performed to determine the mechanical properties of synthetic copolymers. Cuboid samples were prepared using melt processing. All samples in the group of PEG 1.5k and PEG 3.1k, and specific samples (PEG 8.5k-A and PEG 8.5k-B), were too fragile to be loaded between the clamps of the tensile failure test equipment. We considered that the mechanical properties of these samples were poor, so we excluded these samples from further tensile tests.

**Figure 8:**
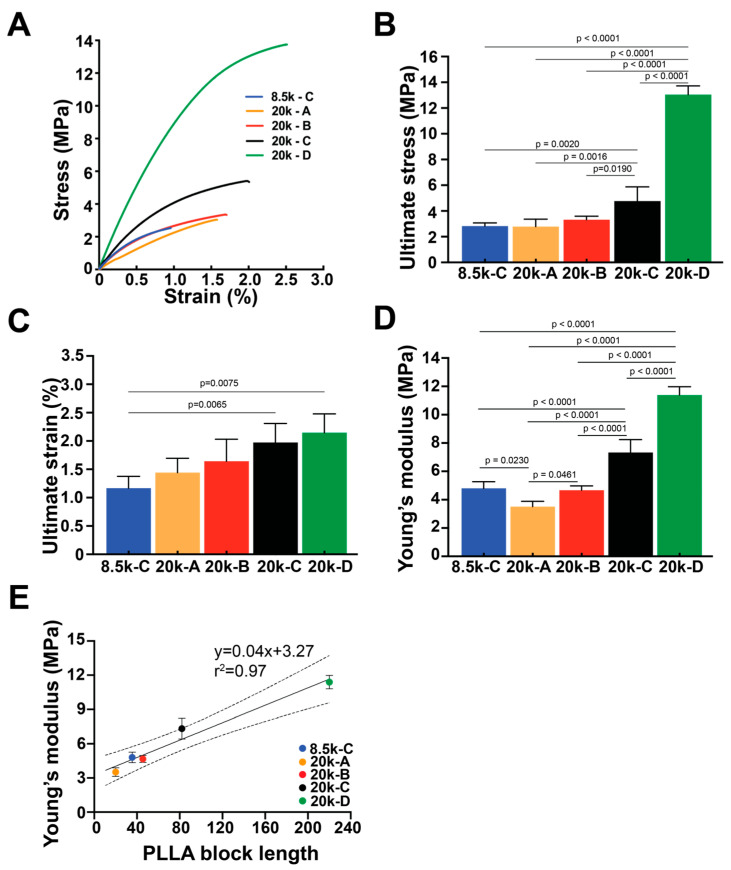
Mechanical properties of triblock PLLA-*b*-PEG-*b*-PLLA copolymers. (**A**) Stress–strain curves. (**B**) Ultimate stress. (**C**) Ultimate strain. (**D**) Young’s modulus of PLLA-PEG-PLLA copolymers with varied PEG molecular weight (8.5 kDa or 20 kDa) and increasing PLLA content ((**A**–**D**), respectively). (**E**) Linear correlation of Young’s modulus as a function of PLLA block length. Two-way ANOVA tests with Tukey’s post hoc test were used. A simple linear regression was used; 95% confidence interval of the best-fit line is shown in dashed lines.

The mechanical properties of the remaining samples (PEG 8.5k-C, PEG 20k-A to D) were investigated. Representative stress–strain curves of each sample are displayed in Figure 8A, showing that increasing PLLA block length contributed to the increase in ultimate stress and strain and Young’s modulus. The stress–strain curve of PEG 8.5k-C was almost overlapping with that of PEG 20k-B, indicating that the elasticity of the copolymer is mainly contributed by the PLLA domain. PEG 20k-D exhibited the highest ultimate stress (13.0 ± 0.67 MPa) and ultimate strain (2.15 ± 0.33%) (Figure 8B,C). Compared to all samples, PEG 20k-A exhibited the lowest Young’s modulus (3.5 ± 0.37 MPa), whereas PEG 20k-D showed the highest Young’s modulus (11.4 ± 0.59 MPa) (Figure 8D). Interestingly, we noticed a linear structure–property relationship between PLLA block length and Young’s modulus (r^2^ = 0.97) using a simple linear regression (Figure 8E) based on empirical data.

## 4. Discussion

PLLA-*b*-PEG-*b*-PLLA copolymers have been widely explored due to their well-defined block compositions and tunable physicochemical properties [8,32]. Herein, we systematically investigated how variations in PEG molecular weight and PLLA block length influence the chemical, thermal, mechanical, and swelling behaviors. We identified empirical correlations of the structure–property relationships and compositional thresholds that govern material performance, providing new insights into the rational design of PLLA-based triblock copolymers. To be clear, Table 3 summarizes the experimental matrix to show which samples were successfully synthesized and characterized and which did not.

The successful synthesis of copolymers was confirmed by ^1^HNMR spectra. Three characteristic peaks of PLLA-PEG-PLLA, with PLLA –CH (~5.3 ppm), PEG –CH_2_ (~3.6 ppm), and PLLA –CH_3_ (~1.58 ppm), were observed. Similar spectra were also observed by previous studies [25,33,34]. The use of different deuterated solvents in ^1^HNMR was able to slightly shift the characteristic peaks. In this study, samples were dissolved in CDCl_3_. The chemical shift of methyl groups of PLLA at ~1.58 ppm observed in this study was noted at ~1.5 ppm, as reported by Rashkov et al. [34], where the solvent was DMSO-d_6_. Two small peaks adjacent to the main PEG methylene protons (~3.6 ppm) may be attributed to the PEG segments near the PEG-PLLA junction, as the local environment of methylene protons differs slightly from that of PEG in the bulk, which was similarly observed by Peng et al. [25].

Phase separation has been observed in block copolymers, where each domain can self-crystallize in triblock copolymers [35]. We selected the PEG 20k-D with the longest PLLA block length and ramped it from 0 to 200 °C in DSC; the characteristic melting peak of PLLA (175–180 °C) [36] was not observed, which was similarly demonstrated by Papeloer et al. [37]. Although we did not try different DSC protocols, as they also affect the melting behavior [38], our experiment showed that no clear PLLA crystalline melting transition was detected under the chosen DSC protocol. Surface characterizations, such as SEM, TEM, AFM, SAXS, or WAXS, would be beneficial to understand the phase separation behavior of these synthetic copolymers.

PLLA had a much higher melting temperature than PEG; however, its influence on the overall thermal behavior of the copolymers diminished with an increasing molecular weight of PEG. We found a linear correlation between composition and melting temperatures, where T_m,onset_ and T_m, peak_ decreased monotonically with increasing PLLA mol%, with a minimum goodness of fit (r^2^) of 0.78 (Figure S2). This decreasing trend may be primarily attributed to the disruption of PLLA blocks to the crystallized PEG, which was also observed in other studies [29,30]. Consistently, the melting enthalpy of the material, ∆H, decreased substantially with reductions of 47.8%, 45.3%, 37.0%, and 34.2% for PEG 1.5k-A, 3.1k-A, 8.5k-A, and 20k-A, respectively. Although PEG 20k-A contained approximately 2.4-fold more PLLA than PEG 8.5k-A, both samples showed comparable ∆H. To account for compositional effects, we normalized ∆H by PEG wt% (∆H_PEG_) of each sample to evaluate the contribution of the PEG domain and calculated the relative crystallinity of PEG domains (X_PEG_) [30]. For each set of copolymers, the sample with the highest ∆H_PEG_ and X_PEG_ was accompanied by the highest melting temperatures. A general decrease in ∆H_PEG_ was correlated with decreasing X_PEG_, indicating that the decrease in ∆H was not attributed solely to compositional dilution but was also associated with disruption of PEG crystallinity. Similar observations are documented by Zhou et al. that increasing PLLA block length dramatically decreased the X_PEG_, independent of PEG architectures [30]. Although PEG wt% decreased in PEG 20k A–C, the X_PEG_ remained in a similar range, which may be attributed to the required PLLA length to disrupt the intrinsic crystallinity of PEG 20K being 82 units. Compared to Zhou et al., who reported that ~194 repeat units of PLLA on each side of PLLA–PEG(10k)-PLLA resulted in a PEG crystallinity value of 0.15 [30], the X_PEG_ values of PEG 20k-D in this study were still higher with PLLA repeat units of ~222.0 on each side. This is likely owing to the crystallinity of PEG increasing with increasing PEG M_n_ [39]. This observation suggests that PEG crystallinity, rather than PLLA content alone, plays a more important role in determining thermal transitions.

PEG M_n_ and the length of PLLA end blocks both affect the mechanical properties of triblock copolymers. In the range of copolymers with low M_n_ PEG (PEG 1.5k and 3.4k), all copolymers were too fragile to form solid samples. Previous studies showed that triblock copolymers with a center block of PEG 2k and 3.5k were formed as hydrogels with a storage modulus in the range of 1000–5000 Pa [40], indicating that copolymers with low M_n_ PEG exhibited relatively poor tensile strength, which is of magnitudes lower than that of the testable samples in this study (ultimate stress: 3.5–11.4 MPa). Thus, the quantitative mechanical analysis applies only to a subset of samples, PEG 8.5k-C and PEG 20k A-D.

Longer PLLA block lengths enable greater chain entanglement, thereby enhancing resistance to deformation and increasing stiffness. Notably, Young’s modulus ranged from 3.5 to 11.4 MPa, consistent with previously reported PLLA-based materials [41,42]. Importantly, we identified an empirical composition-dependent relationship in which a minimum ~4-fold increase in PLLA block length resulted in statistically distinguishable mechanical properties within the PEG 20k system. Samples with different PEG molecular weights but a comparable number of PLLA units (e.g., PEG 8.5k-C vs. PEG 20k-B) exhibited similar tensile behavior, reinforcing that PLLA block length is the primary determinant of mechanical performance. These results suggest that mechanical properties in the current PLLA-PEG-PLLA triblock copolymer system can be tuned predictably through the modulation of PLLA block length. The empirical dependence on PLLA content highlights its major role in governing network integrity and stiffness. Additionally, the PLLA domain in this study is likely amorphous, which is attributed to the rapid thermal processing conditions. Compared to other processing methods involving prolonged annealing [43,44], the short melting and cooling timeline (~30 min total) likely limited crystalline domain formation, resulting in reduced strain at break (~2.5%) across all samples.

Increasing repeat units of PLLA reduced the swelling ratio of the copolymers, despite the hydrophilic nature of PEG domains. This trend indicates that swelling behavior is governed by the balance between hydrophilic PEG domains and hydrophobic PLLA domains, with increasing PLLA content systematically suppressing water uptake. In the current system, we showed that a minimum PLLA block length (~20 repeat units) was an approximate empirical boundary under the current test conditions to maintain structural integrity during swelling, regardless of the molecular weights of PEG. These findings suggest that PLLA content plays a major role in stabilizing the copolymer network against dissolution, providing a compositional threshold for balanced hydration and structural integrity.

## 5. Conclusions

A range of PEG molecular weights was utilized to assess the influence of the PEG center blocks and PLLA side blocks on triblock copolymer properties. Under the specific synthesis and processing conditions used in the study, PLLA-*b*-PEG-*b*-PLLA triblock copolymers were successfully synthesized with tunable physical, thermal, mechanical, and swelling behaviors. Varying the mass of initial monomers, the size, molecular weight, melting points, enthalpies, stress, strain, and Young’s modulus were characterized. Linear correlations of melting temperatures and Young’s modulus were found to be a function of copolymer composition using empirical data, suggesting that these parameters can be systematically controlled under the current experimental conditions. Copolymers composed of PEG with molecular weights of less than 8.5 kDa showed suboptimal thermal, mechanical, and swelling performances. An empirical compositional threshold was found such that a minimum PEG molecular weight of 8.5 kDa was required for mechanical behavior. An approximate minimum of 20 repeat units of PLLA was required to maintain structural integrity during swelling.

## Figures and Tables

**Table 1:** Composition of PLLA-*b*-PEG-*b*-PLLA triblock copolymers.

Sample ID	Copolymers	^1^H NMR					GPC	
		M_n_ (Da)	PLLA M_n_ % ^	M_n_ Ratio: PEG/PLLA	PLLA Block Length	PEG wt%	M_n_ (Da)	PDI
1.5k *	PEG_33_	N/A	N/A	N/A	N/A	100%	1450 ± 2.0	1.05
1.5k-A	PLLA_1.7_-PEG_33_-PLLA_1.7_	1690	14.1%	6.1:1	1.7	85.8%	1780 ± 3.0	1.09
1.5k-B	PLLA_4.0_-PEG_33_-PLLA_4.0_	2020	28.9%	2.6:1	4.0	71.8%	2000 ± 0.5	1.07
1.5k-C	PLLA_10.2_-PEG_33_-PLLA_10.2_	2912	50.2%	0.99:1	8.6	49.8%	2550 ± 2.0	1.11
3.1k *	PEG_70_	N/A	N/A	N/A	N/A	100%	3140 ± 0.5	1.05
3.1k-A	PLLA_4.4_-PEG_71_-PLLA_4.4_	3633	13.6%	6.4:1	3.4	86.4%	3500 ± 8.0	1.07
3.1k-B	PLLA_8.0_-PEG_71_-PLLA_8.0_	3900	26.8%	2.7:1	8.0	80.5%	4030 ± 2.0	1.09
3.1k-C	PLLA_16.6_-PEG_71_-PLLA_16.6_	4446	43.1%	1.3:1	16.6	70.7%	4630 ± 11.0	1.07
8.5k *	PEG_193_	N/A	N/A	N/A	N/A	100%	8480 ± 76.0	1.08
8.5k-A	PLLA_8.4_-PEG_193_-PLLA_8.4_	9205	13.9%	6.6:1	8.4	92.2%	9240 ± 47.0	1.08
8.5k-B	PLLA_15.6_-PEG_193_-PLLA_15.6_	10,253	22.0%	3.6:1	15.6	82.7%	10,020 ± 30.0	1.09
8.5k-C	PLLA_35.3_-PEG_193_-PLLA_35.3_	13,080	38.8%	1.6:1	35.3	64.8%	10,860 ± 59.0	1.15
20k *	PEG_452_	N/A	N/A	N/A	N/A	100%	19,900 ± 1630	1.10
20k-A	PLLA_20.0_-PEG_452_-PLLA_20.0_	22,880	12.6%	6.9:1	20.0	87.0%	22,620 ± 1400	1.12
20k-B	PLLA_45.5_-PEG_452_-PLLA_45.5_	26,550	24.7%	3.1:1	45.5	75.0%	22,300 ± 1120	1.14
20k-C	PLLA_82.0_-PEG_452_-PLLA_82.0_	31,790	37.9%	1.7:1	82.0	62.6%	28,500 ± 1820	1.24
20k-D	PLLA_220.0_-PEG_452_-PLLA_222.0_	51,543	61.2%	0.6:1	222.0	38.6%	N/A	N/A

* PEG block length was calculated using the M_n_ of PEG (from GPC) divided by the theoretical molecular weight of PEG (44 g/mol) in the copolymer. PLLA block length was calculated in the same way using a repeat unit molecular weight of 72 g/mol. ^ PLLA M_n_% was calculated by the division of the M_n_ of PLLA over the M_n_ of copolymer using ^1^H NMR results.

**Table 2:** T_m,onset_, T_m,peak_, and melting enthalpies of triblock PLLA-*b*-PEG-*b*-PLLA copolymers.

**Sample ID**	**T_m,onset_ (°C)**	**T_m,peak_ (°C)**	**∆H (J/g)**	**∆H_PEG_ (J/g)**
PEG 1.5k	37.6 ± 0.6	51.6 ± 2.3	122.4 ± 0.6	122.4
PEG 1.5k-A	24.8 ± 0.9	36.7 ± 0.3	63.8 ± 1.9	74.4
PEG 1.5k-B	20.6 ± 0.6	32.4 ± 0.3	47.4 ± 2.3	66.0
PEG 1.5k-C	13.2 ± 0.3	24.3 ± 0.9	24.8 ± 0.9	49.8
**Sample ID**	**T_m,onset_ (°C)**	**T_m,peak_ (°C)**	**∆H (J/g)**	**∆H_PEG_ (J/g)**
PEG 3.1k	53.7 ± 0.2	61.8 ± 0.5	136.1 ± 0.3	136.1
PEG 3.1k-A	40.2 ± 0.6	46.1 ± 0.3	74.4 ± 0.5	86.1
PEG 3.1k-B	37.3 ± 1.5	44.5 ± 0.6	65.7 ± 1.4	81.6
PEG 3.1k-C	33.6 ± 0.1	41.3 ±0.2	42.7 ± 1.4	60.4
**Sample ID**	**T_m,onset_ (°C)**	**T_m,peak_ (°C)**	**∆H (J/g)**	**∆H_PEG_ (J/g)**
PEG 8.5k	58.6 ± 0.1	66.2 ± 0.1	148.0 ± 3.0	148.0
PEG 8.5k-A	49.7 ± 0.4	56.4 ± 0.4	93.2 ± 3.0	101.1
PEG 8.5k-B	47.2 ± 0.3	54.1 ± 0.2	75.2 ± 1.7	90.9
PEG 8.5k-C	43.3 ± 0.4	51.3 ± 0.5	57.2 ± 2.2	88.3
**Sample ID**	**T_m,onset_ (°C)**	**T_m,peak_ (°C)**	**∆H (J/g)**	**∆H_PEG_ (J/g)**
PEG 20k	61.3 ± 0.7	68.0 ± 0.1	140.8 ± 3.8	140.8
PEG 20k-A	55.6 ± 0.6	60.0 ± 0.4	92.6 ± 0.5	106.4
PEG 20k-B	54.3 ± 0.1	59.2 ± 0.2	76.0 ± 1.3	101.3
PEG 20k-C	52.1 ± 0.7	57.5 ± 0.5	68.4 ± 1.3	109.3
PEG 20k-D	48.6 ± 1.0	54.5 ± 0.5	33.3 ± 4.0	86.3

∆H: Melting enthalpy of copolymer determined by DSC. ∆H_PEG__:_ Melting enthalpy normalized to PEG wt%.

**Table 3:** Summary of full experimental matrix.

Sample ID	Synthesis	GPC	DSC	Tensile Test	Swelling
PEG 1.5k	N/A	Y	Y	N/A	N/A
PEG 1.5k-A	Y	Y	Y	N/A	N/A
PEG 1.5k-B	Y	Y	Y	N/A	N/A
PEG 1.5k-C	Y	Y	Y	N/A	N/A
PEG 3.1k	N/A	Y	Y	N/A	N/A
PEG 3.1k-A	Y	Y	Y	N/A	N/A
PEG 3.1k-B	Y	Y	Y	N/A	N/A
PEG 3.1k-C	Y	Y	Y	N/A	N/A
PEG 8.5k	N/A	Y	Y	N/A	N/A
PEG 8.5k-A	Y	Y	Y	N/A	N/A
PEG 8.5k-B	Y	Y	Y	N/A	N/A
PEG 8.5k-C	Y	Y	Y	Y	Y
PEG 20k	N/A	Y	Y	N/A	N/A
PEG 20k-A	Y	Y	Y	Y	N/A
PEG 20k-B	Y	Y	Y	Y	Y
PEG 20k-C	Y	Y	Y	Y	Y
PEG 20k-D	Y	N/A	Y	Y	Y

Y: This sample is successfully synthesized, characterized thermally or mechanically, remained intact after swelling. N/A: This sample failed under this characterization.

## Data Availability

The original contributions presented in this study are included in the article/Supplementary Material. Further inquiries can be directed to the corresponding author.

## Supplementary Materials

The following supporting information can be downloaded at https://www.mdpi.com/article/10.3390/polym18091127/s1. Figure S1. DSC thermograms all samples. (A) PEG 1.5k A-C. (B) PEG 3.1k A-C. (C) PEG 8.5k A-C. (D) PEG 20k A-C. (E) PEG 20k-D Exothermal direction is up. Figure S2. Linear regression of Tm, onset (magenta line with open black circle) and Tm, peak (brown line with closed black circle) as a function of PLLA mol % based on pure PEG molecular weight. (A) 1.5k copolymers; (B) 3.1k copolymers; (C) 8.5k copolymers; (D) 20k copolymers.

## References

[1] Peppas N.A., Langer R. (1994). New challenges in biomaterials. Science.

[2] Langer R. (2000). Biomaterials in drug delivery and tissue engineering: One laboratory’s experience. Acc. Chem. Res..

[3] Adams M.L., Lavasanifar A., Kwon G.S. (2003). Amphiphilic block copolymers for drug delivery. J. Pharm. Sci..

[4] Cao D., Ding J. (2022). Recent advances in regenerative biomaterials. Regen. Biomater..

[5] Kubisa P., Lapienis G., Biela T. (2021). Star-shaped copolymers with PLA–PEG arms and their potential applications as biomedical materials. Polym. Adv. Technol..

[6] Rostami N., Faridghiasi F., Ghebleh A., Noei H., Samadzadeh M., Gomari M.M., Tajiki A., Abdouss M., Aminoroaya A., Kumari M. (2023). Design, synthesis, and comparison of PLA-PEG-PLA and PEG-PLA-PEG copolymers for curcumin delivery to cancer cells. Polymers.

[7] Mikos A.G., Sarakinos G., Leite S.M., Vacant J.P., Langer R. (1993). Laminated three-dimensional biodegradable foams for use in tissue engineering. Biomaterials.

[8] Capuana E., Lopresti F., Ceraulo M., La Carrubba V. (2022). Poly-l-Lactic Acid (PLLA)-Based Biomaterials for Regenerative Medicine: A Review on Processing and Applications. Polymers.

[9] Casalini T., Rossi F., Castrovinci A., Perale G. (2019). A Perspective on Polylactic Acid-Based Polymers Use for Nanoparticles Synthesis and Applications. Front. Bioeng. Biotechnol..

[10] Mest D., Humble G. (2010). Review and Evaluation of Treatment Procedures Using Injectable Poly-L-Lactic Acid in the Treatment of Human Immunodeficiency Virus-associated Facial Lipoatrophy. J. Clin. Aesthetic Dermatol..

[11] Ao Y.J., Yi Y., Wu G.H. (2024). Application of PLLA (Poly-L-Lactic acid) for rejuvenation and reproduction of facial cutaneous tissue in aesthetics: A review. Medicine.

[12] Liu S., Yu J., Li H., Wang K., Wu G., Wang B., Liu M., Zhang Y., Wang P., Zhang J. (2020). Controllable Drug Release Behavior of Polylactic Acid (PLA) Surgical Suture Coating with Ciprofloxacin (CPFX)-Polycaprolactone (PCL)/Polyglycolide (PGA). Polymers.

[13] D’souza A.A., Shegokar R. (2016). Polyethylene glycol (PEG): A versatile polymer for pharmaceutical applications. Expert Opin. Drug Deliv..

[14] Cleveland M.V., Flavin D.P., Ruben R.A., Epstein R.M., Clark G.E. (2001). New polyethylene glycol laxative for treatment of constipation in adults: A randomized, double-blind, placebo-controlled study. South. Med. J..

[15] Christoforou I., Kalatzis A., Siamidi A., Vlachou M., Pispas S., Pippa N. (2025). The Ubiquitous Use of Polyethylene Glycol in Pharmaceutical Design and Development: Technological Aspects and Future Perspectives. Nanomaterials.

[16] Wilding K.M., Smith A.K., Wilkerson J.W., Bush D.B., Knotts T.A., Bundy B.C. (2018). The locational impact of site-specific PEGylation: Streamlined screening with cell-free protein expression and coarse-grain simulation. ACS Synth. Biol..

[17] Santhanakrishnan K.R., Koilpillai J., Narayanasamy D. (2024). PEGylation in Pharmaceutical Development: Current Status and Emerging Trends in Macromolecular and Immunotherapeutic Drugs. Cureus.

[18] Srinivasan S., Manoj V. (2021). A Decade of Effective Dry Eye Disease Management with Systane Ultra (Polyethylene Glycol/Propylene Glycol with Hydroxypropyl Guar) Lubricant Eye Drops. Clin. Ophthalmol..

[19] Menees S.B., Lembo A.J., Chey W.D. (2022). Polyethylene Glycol 3350 in the Treatment of Chronic Idiopathic Constipation: Post hoc Analysis Using FDA Endpoints. Can. J. Gastroenterol. Hepatol..

[20] Lian R., Zhou D., Xiao L., Rodrigues J., Sheng R., Bai Z., Li Y., Liu C. (2025). PLLA–PEG/mPEG Copolymer with Improved Hydrophilicity, Crystallinity, and Biocompatibility: An In-Depth Study on the Crystallization Kinetics. ACS Appl. Mater. Interfaces.

[21] Bikiaris N.D., Malini E., Christodoulou E., Klonos P.A., Kyritsis A., Galaris A., Pantopoulos K. (2025). Amphiphilic Thermoresponsive Triblock PLA-PEG-PLA and Diblock mPEG-PLA Copolymers for Controlled Deferoxamine Delivery. Gels.

[22] Li L., Cao Z.-Q., Bao R.-Y., Xie B.-H., Yang M.-B., Yang W. (2017). Poly(l-lactic acid)-polyethylene glycol-poly(l-lactic acid) triblock copolymer: A novel macromolecular plasticizer to enhance the crystallization of poly(l-lactic acid). Eur. Polym. J..

[23] Yun X., Li X., Jin Y., Sun W., Dong T. (2018). Fast Crystallization and Toughening of Poly(L-lactic acid) by Incorporating with Poly(ethylene glycol) as a Middle Block Chain. Polym. Sci. Ser. A.

[24] Xie W., Jiang C., Yu X., Shi X., Wang S., Sun Y., Yin M., Wu D. (2021). Stereocomplex-Induced Self-Assembly of PLLA-PEG-PLLA and PDLA-PEG-PDLA Triblock Copolymers in an Aqueous System. ACS Appl. Polym. Mater..

[25] Peng H., Varanasi S., Wang D.K., Blakey I., Rasoul F., Symons A., Hill D.J.T., Whittaker A.K. (2016). Synthesis, swelling, degradation and cytocompatibility of crosslinked PLLA-PEG-PLLA networks with short PLLA blocks. Eur. Polym. J..

[26] Wang D., Hill D.J.T., Peng H., Symons A., Varanasi S., Whittaker A.K., Rasoul F. (2010). Development of Injectable Biodegradable Multi-Arms PEG-Based Hydrogels: Swelling and Degradation Investigations. Macromol. Symp..

[27] Cui H., Shao J., Wang Y., Zhang P., Chen X., Wei Y. (2013). PLA-PEG-PLA and Its Electroactive Tetraaniline Copolymer as Multi-interactive Injectable Hydrogels for Tissue Engineering. Biomacromolecules.

[28] Mao H., Wang C., Chang X., Cao H., Shan G., Bao Y., Pan P. (2018). Poly(lactic acid)/poly(ethylene glycol) stereocomplexed physical hydrogels showing thermally-induced gel–sol–gel multiple phase transitions. Mater. Chem. Front..

[29] Gong Y., Song W., Wu Y., Zhang D., Liu Y., Zhao Q., He M., Chen X. (2020). Effect of chain segment length on crystallization behaviors of poly(l-lactide-b-ethylene glycol-b-l-lactide) triblock copolymer. Polym. Polym. Compos..

[30] Zhou D., Shao J., Li G., Sun J., Bian X., Chen X. (2015). Crystallization behavior of PEG/PLLA block copolymers: Effect of the different architectures and molecular weights. Polymer.

[31] Wu Y., Li L., Chen S., Qin J., Chen X., Zhou D., Wu H. (2020). Synthesis, characterization, and crystallization behaviors of poly(D-lactic acid)-based triblock copolymer. Sci. Rep..

[32] Wi J., Choi J., Lee S.-H. (2026). PLA-Based Biodegradable Polymer from Synthesis to the Application. Polymers.

[33] Ghasemi R., Abdollahi M., Emamgholi Zadeh E., Khodabakhshi K., Badeli A., Bagheri H., Hosseinkhani S. (2018). mPEG-PLA and PLA-PEG-PLA nanoparticles as new carriers for delivery of recombinant human Growth Hormone (rhGH). Sci. Rep..

[34] Rashkov I., Manolova N., Li S.M., Espartero J.L., Vert M. (1996). Synthesis, Characterization, and Hydrolytic Degradation of PLA/PEO/PLA Triblock Copolymers with Short Poly(l-lactic acid) Chains. Macromolecules.

[35] Buttaro L.M., Drufva E., Frey M.W. (2014). Phase separation to create hydrophilic yet non-water soluble PLA/PLA-b-PEG fibers via electrospinning. J. Appl. Polym. Sci..

[36] Dillon B., Doran P., Fuenmayor E., Healy A.V., Gately N.M., Major I., Lyons J.G. (2019). Influence of Annealing and Biaxial Expansion on the Properties of Poly(l-Lactic Acid) Medical Tubing. Polymers.

[37] Papeloer Q., Van Velthem P., Demoustier-Champagne S., Jonas A.M. (2024). Phase Diagram of Semicrystalline Blends of Poly(l-lactic acid) and Poly(ethylene glycol). ACS Appl. Polym. Mater..

[38] Zhou D., Sun J., Shao J., Bian X., Huang S., Li G., Chen X. (2015). Unusual crystallization and melting behavior induced by microphase separation in MPEG-b-PLLA diblock copolymer. Polymer.

[39] Pielichowski K., Flejtuch K. (2002). Differential scanning calorimetry studies on poly(ethylene glycol) with different molecular weights for thermal energy storage materials. Polym. Adv. Technol..

[40] Abebe D.G., Fujiwara T. (2012). Controlled Thermoresponsive Hydrogels by Stereocomplexed PLA-PEG-PLA Prepared via Hybrid Micelles of Pre-Mixed Copolymers with Different PEG Lengths. Biomacromolecules.

[41] Ciapetti G., Granchi D., Devescovi V., Baglio S.R., Leonardi E., Martini D., Jurado M.J., Olalde B., Armentano I., Kenny J.M. (2012). Enhancing osteoconduction of PLLA-based nanocomposite scaffolds for bone regeneration using different biomimetic signals to MSCs. Int. J. Mol. Sci..

[42] Wu H., Lei P., Liu G., Shrike Zhang Y., Yang J., Zhang L., Xie J., Niu W., Liu H., Ruan J. (2017). Reconstruction of Large-scale Defects with a Novel Hybrid Scaffold Made from Poly(L-lactic acid)/Nanohydroxyapatite/Alendronate-loaded Chitosan Microsphere: In vitro and in vivo Studies. Sci. Rep..

[43] Pan P., Kai W., Zhu B., Dong T., Inoue Y. (2007). Polymorphous Crystallization and Multiple Melting Behavior of Poly(l-lactide):  Molecular Weight Dependence. Macromolecules.

[44] Pan P., Zhu B., Kai W., Dong T., Inoue Y. (2008). Polymorphic Transition in Disordered Poly(l-lactide) Crystals Induced by Annealing at Elevated Temperatures. Macromolecules.

