# Supplementary material for: Empirical Structure–Property Relationships of PLLA-b-PEG-b-PLLA Triblock Copolymers with Tunable Thermal, Tensile, and Swelling Behavior
Source: Polymers (Basel). 2026 May 2;18(9):1127. doi: 10.3390/polym18091127 (PMC13165789; doi:10.3390/polym18091127)
Supplement: Supplementary file 1 [file polymers-18-01127-s001.zip › polymers-4275792-supplementary.pdf]

Supplemental information

Empirical Structure–Property Relationships of PLLA-*b*-PEG-*b*-PLLA Triblock Copolymers with Tunable Thermal, Tensile, and Swelling Behavior

Yang Hu[1], Xiaoya Sun[2], Wei Wu[1], Adam K. Ekenseair[1,*]

[1] Department of Chemical Engineering, College of Engineering, Northeastern University, Boston, MA 02115, USA;
hu.yang@northeastern.edu (Y.H.); wu.wei@northeastern.edu (W.W.)
[2] Department of Chemistry and Chemical Biology, College of Science, Northeastern University, Boston, MA 02115, USA; sun.xia@northeastern.edu (X.S.)
***** Correspondence: a.ekenseair@northeastern.edu

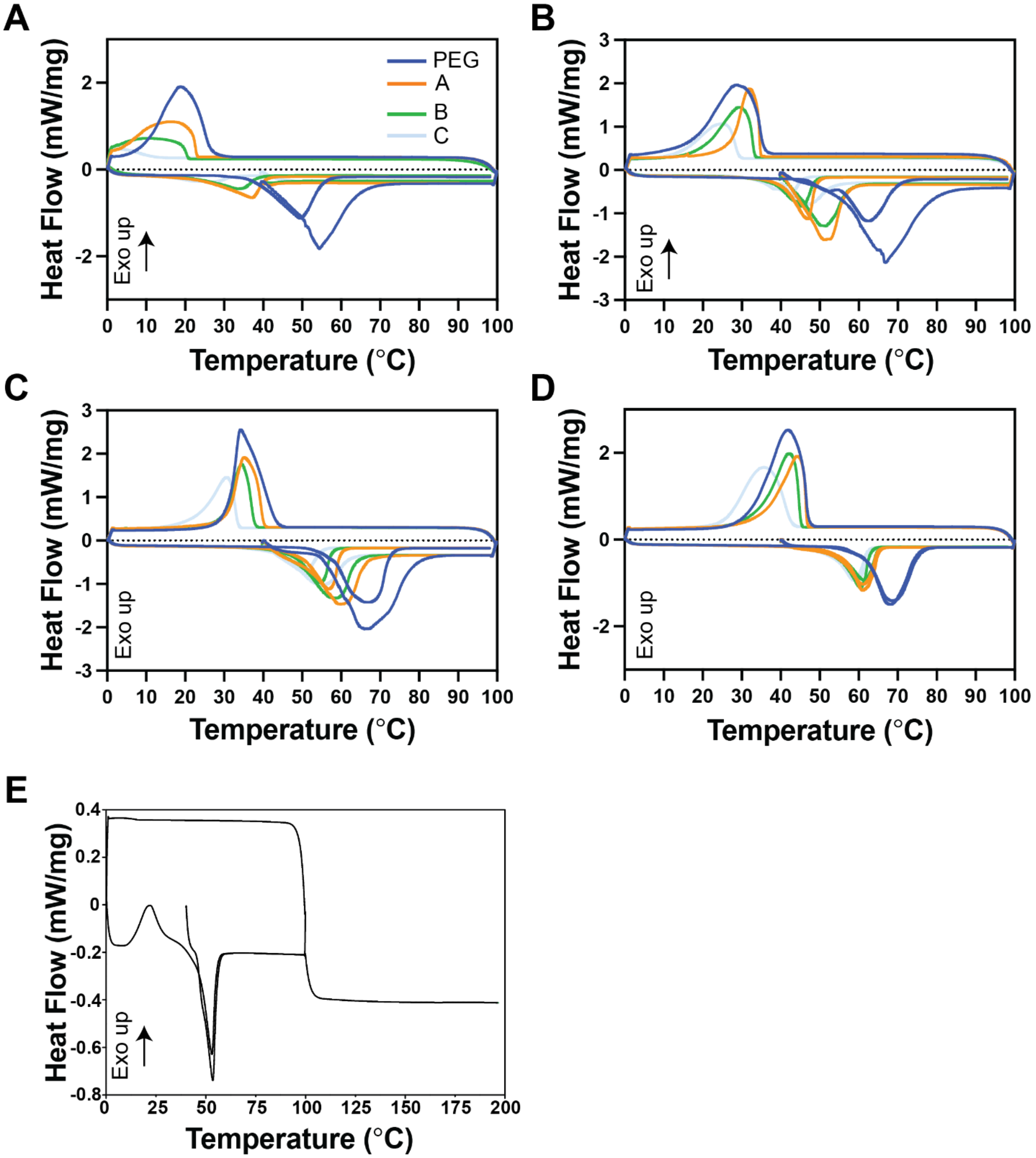

Figure S1. DSC thermograms all samples. (A) PEG 1.5k A-C. (B) PEG 3.1k A-C. (C) PEG 8.5k A-C. (D) PEG 20k A-C. (E) PEG 20k-D Exothermal direction is up.

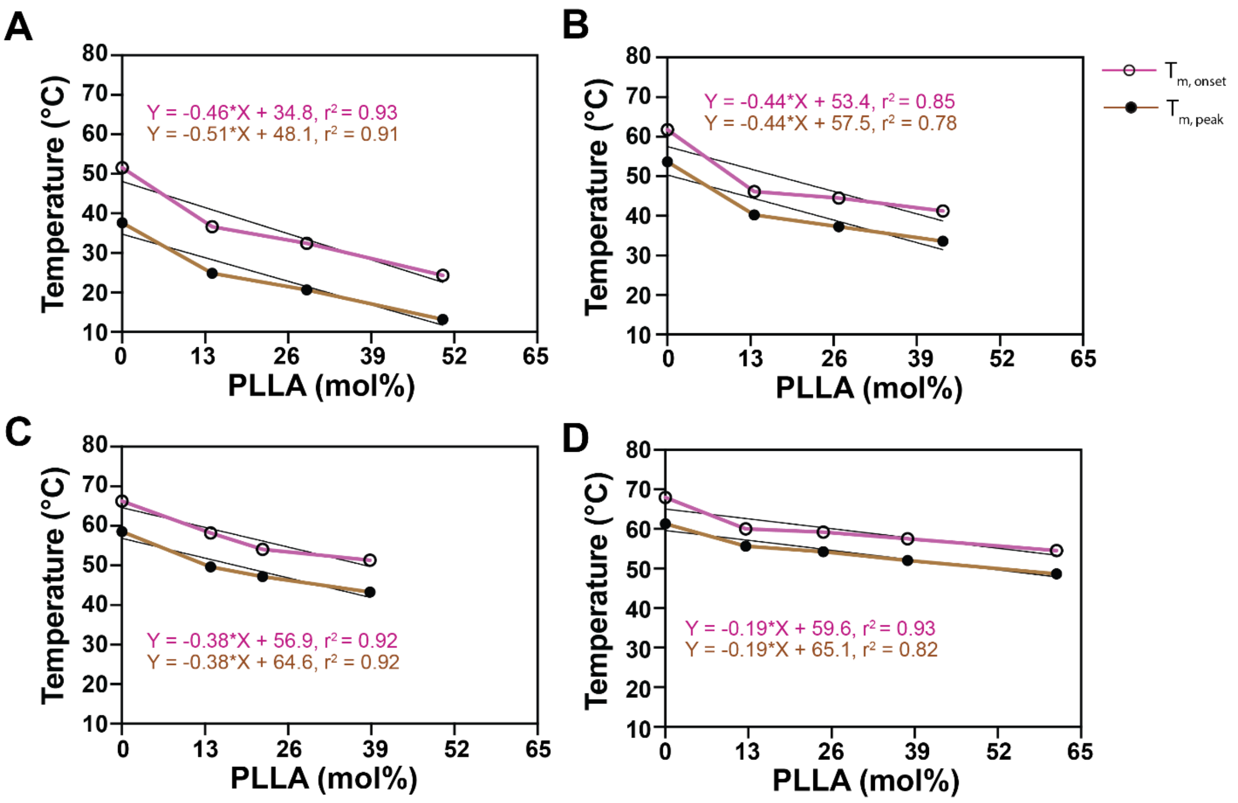

Figure S2. Linear regression of $T_{m,\ onset}$ (magenta line with open black circle) and $T_{m,\ peak}$ (brown line with closed black circle) as a function of PLLA mol % based on pure PEG molecular weight. (A) 1.5k copolymers; (B) 3.1k copolymers; (C) 8.5k copolymers; (D) 20k copolymers.
